# Telomere length is causally connected to brain MRI image derived phenotypes: A mendelian randomization study

**DOI:** 10.1371/journal.pone.0277344

**Published:** 2022-11-18

**Authors:** Ahmed Salih, Ilaria Boscolo Galazzo, Steffen E. Petersen, Karim Lekadir, Petia Radeva, Gloria Menegaz, André Altmann

**Affiliations:** 1 Department of Computer Science, University of Verona, Verona, Italy; 2 William Harvey Research Institute, NIHR Barts Biomedical Research Centre, Queen Mary University of London, London, United Kingdom; 3 Barts Heart Centre, St Bartholomew’s Hospital, Barts Health NHS Trust, London, United Kingdom; 4 Dept. de Matemàtiques i Informàtica, University of Barcelona, Barcelona, Spain; 5 Centre for Medical Image Computing (CMIC), Department of Medical Physics and Biomedical Engineering, University College London, London, United Kingdom; University of Maryland at College Park, UNITED STATES

## Abstract

Recent evidence suggests that shorter telomere length (TL) is associated with neuro degenerative diseases and aging related outcomes. The causal association between TL and brain characteristics represented by image derived phenotypes (IDPs) from different magnetic resonance imaging (MRI) modalities remains unclear. Here, we use two-sample Mendelian randomization (MR) to systematically assess the causal relationships between TL and 3,935 brain IDPs. Overall, the MR results suggested that TL was causally associated with 193 IDPs with majority representing diffusion metrics in white matter tracts. 68 IDPs were negatively associated with TL indicating that longer TL causes decreasing in these IDPs, while the other 125 were associated positively (longer TL leads to increased IDPs measures). Among them, ten IDPs have been previously reported as informative biomarkers to estimate brain age. However, the effect direction between TL and IDPs did not reflect the observed direction between aging and IDPs: longer TL was associated with decreases in fractional anisotropy and increases in axial, radial and mean diffusivity. For instance, TL was positively associated with radial diffusivity in the left perihippocampal cingulum tract and with mean diffusivity in right perihippocampal cingulum tract. Our results revealed a causal role of TL on white matter integrity which makes it a valuable factor to be considered when brain age is estimated and investigated.

## Introduction

Telomeres are DNA-protein complexes which protect the end of chromosomes from fusion and degradation. Telomere length (TL) shortens with time (i.e., during each cell cycle) in most human cell types [1]. Among many phenotypes, TL is associated with tumors in the central nervous system [2]. Furthermore, TL is considered as a potential biomarker of aging-related diseases such as Alzheimer’s disease (AD) [3]. While several studies [4, 5] highlighted correlations between TL and brain image derived phenotypes (IDPs), no study has established the causative link between TL and IPDs, yet. In fact, in current studies reduced TL may act as a biological proxy for aging and thus induce a correlation between natural aging and brain integrity. The causal link in this respect could be established by employing Mendelian randomization (MR), a method that leverages results from large Genome-wide association studies (GWAS) to infer causality between exposure and outcome. Thus far, GWAS have identified dozens of single nucleotide polymorphisms (SNPs) with a significant association to TL [1, 6]. These SNPs were found to play a critical role as regulators of leukocyte TL through different mechanisms including deoxynucleoside monophosphate biosynthesis [6] and telomere elongation helicase [7]. Furthermore, these SNPs were used as instruments in previous MR studies to causally associate TL shortening with increasing facial skin aging [8], increased risk of AD [3] and coronary heart disease [1]. Here we used MR to study the causative link between TL and the brain’s micro and macro structure. Knowing this causative landscape will help us to better understand the causal association between shorter TL and development of brain aging-related diseases such as AD. In addition, this study might reveal the causes behind the changes of brain functions and structures during healthy brain aging.

## Results

**Fig 1:**
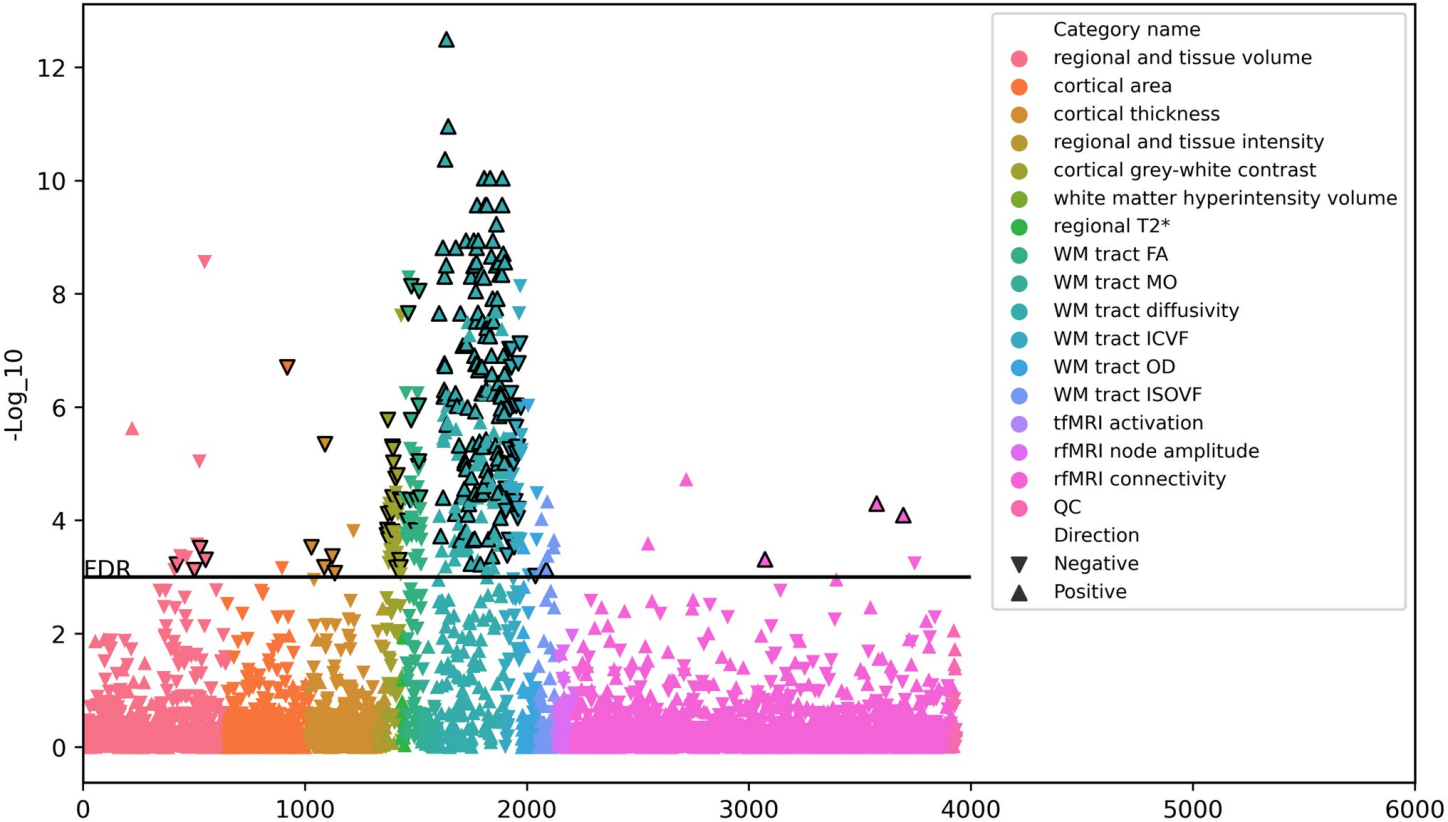
The causal association of TL and brain IDPs using the IVW method. The y-axis represents the −log_10_(*p*−values) of the association. The color of each IDP indicates the MRI modality and the triangle shape indicates whether the identified association (IVW *β* value) is positive (△) or negative (▽). The black horizontal line indicates the FDR-adjusted significance threshold (*P* = 0.004409). The triangles with black border highlight the 193 IDPs that were significantly associated with TL using the IVW method as well as the complementary MR analyses. WM: white matter; FA: fractional anisotropy; MO: diffusion tensor mode; OD: orientation dispersion; ICVF: intracellular volume fraction; ISOVF: isotropic volume fraction; tfMRI: task fMRI; rfMRI: resting-state fMRI; QC: quality control.

Fig 1 shows the result of the MR causality screen between TL and brain IDPs using the inverse variance weighted (IVW) method. Out of the 3,935 tested IDPs, 347 remained statistically significant after adjusting for multiple testing using the FDR method (*P*_FDR_ < 0.05;*P* < 0.004409). However, 119 IDPs were not marginally significant (*P* > 0.05) in the complementary MR analyses (i.e., the weighted mode and weighted median) leaving 228 IDPs. MR-PRESSO [9] was used to investigate effects of pleiotropy in these MR results. Further 35 IDPs were excluded since MR-PRESSO detected horizontal pleiotropy (MR-PRESSO global test *P* < 0.05), which, after SNP outlier removal, was no longer significant at the FDR-corrected p-value threshold. Thus, the final number of the significant IDPs was 193. P-values of MR Egger-intercept of the 193 IDPs indicate no significant pleiotropy (*P* > 0.05). Therefore, overall, 193 out of 3,935 IDPs showed evidence of being significantly influenced by TL, the majority of which are diffusion metrics in different region of interests.

The majority of the significantly associated IDPs (162 of 193) corresponds to different indices from diffusion MRI covering a wide range of white matter tracts (Table 1). Three IDPs correspond to resting-state fMRI and the remaining 28 were derived from T1-weighted MRI, with the majority representing gray-white matter intensity contrasts. The direction of association was uniform for each of the modalities: FA, ICVF and gray-white matter intensity contrast were negatively associated with TL (i.e., longer TL causes decreases); axial (L1), radial (L2, L3) and mean diffusivity (MD) were positively associated with TL (i.e., longer TL causes increases in these values). A full list detailing all the results is available in S1 Table.

**Table 1:** The significant IDPs categorized by modality. ED: effect direction whether it is positive (+) or negative (-).

Category	Number	ED	Category	Number	ED
FA	12	-	ISOVF	1	-
ICVF	27	-	wg intensity-contrast	18	+
L1	18	+	Thickness	5	-
L2	29	+	Area	1	-
L3	42	+	Volume	4	-
MD	32	+	rs-fMRI	3	+
OD	1	-			

**Fig 2:**
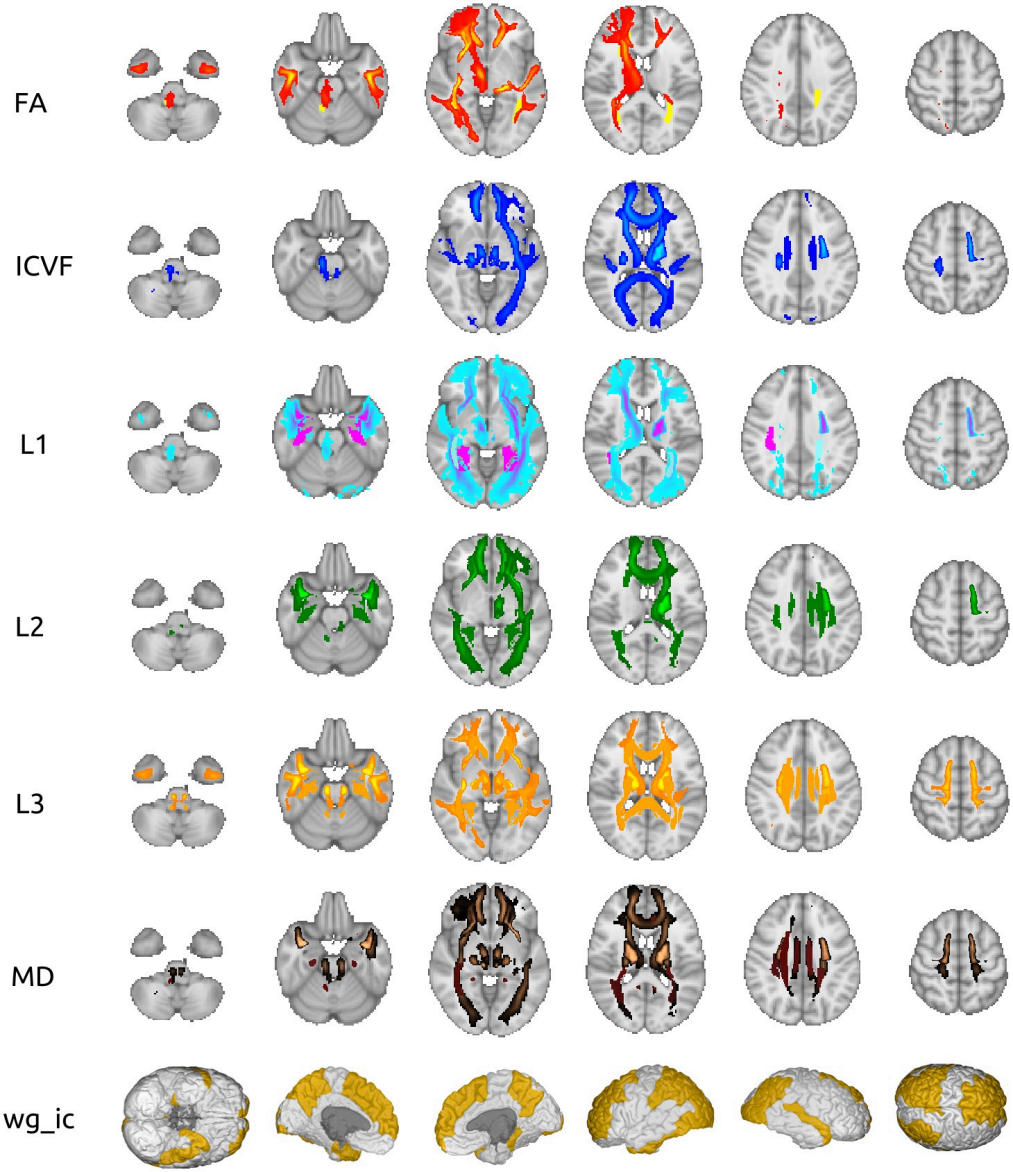
Visual representation of the significant IDPs among the seven most prevalent measures. For the six diffusion indices (top six rows) the tracts that are significantly associated with TL are highlighted. The last row shows the cortical regions with a significant effect of TL on gray-white matter intensity contrast. Different colors within a diffusion measure relate to IDPs extracted from two different methods: tract-based spatial statistics (solid colors) and probabilistic tractography (color gradients). The plots were generated by BrainPainter [10] and FSL [11].

Fig 2 illustrates for the most prevalent diffusion indices the tracts that are causally influenced by TL according to the MR analysis. Many tracts are found to be associated across the various diffusion indices. For instance, almost all diffusion indices were significant in tracts like posterior thalamic radiation and anterior thalamic radiation in both hemispheres. Furthermore, the grey-white matter intensity contrast in many cortical regions were causally associated with TL (Fig 2, S1 Table).

## Discussion

In this study, we performed casual association of TL and 3,935 brain IDPs using MR. The results indicate that TL casually affect 193 brain IDPs. Interestingly, the majority of the significant IDPs were related to white matter but not to gray matter. Even the measure with the highest number of significant IDPs derived from T1-weighted MRI was the grey-white matter intensity contrast. In the context of aging the diffusion indices can be interpreted in terms of white matter integrity. For instance, high FA values suggest increased diffusion directionality and thereby higher white matter integrity. Contrary, high MD values suggest a higher average rate of diffusion and thus impaired WM integrity [12]. Therefore, with increasing age, FA tends to decline while MD tends to increase in white matter tracts. In previous works some of the IDPs were also identified as informative features to model and estimate brain age. For instance, ten out of the 193 IDPs were previously reported to have a significant association with brain age delta [13] that are: weighted mean anterior thalamic radiation left (L2 and L3), weighted mean posterior thalamic radiation left (L2 and MD), weighted mean uncinate fasciculus left (MD), weighted mean posterior thalamic radiation right (L2 and MD), weighted mean anterior thalamic radiation right (MD and L3) and TBSS external capsule right (ICVF). Moreover, three other IDPs (i.e., TBSS posterior thalamic radiation right (L3), weighted mean uncinate fasciculus left (L3) and TBSS cingulum hippocampus right (MD)) were also previously reported to be important to estimate brain age [14]. However, despite changes in these IDPs having been reported before as potential biomarkers of brain aging, the driving factor behind these changes was missing. Our MR analysis demonstrated that TL is one key factor that influences the observed values of these IDPs, and by extension brain aging.

Previous studies demonstrated that FA and ICVF decline with aging while axial, radial and MD increase [15], although regional differences have been described [16, 17]. Based on these findings and considering TL as a proxy for cellular aging, we would expect a positive correlations between TL and FA/ICVF as well as negative correlations between TL and axial, radial and mean diffusivity. However, the MR results support the opposite direction, indicating decreasing FA and ICVF with increasing TL as well as increasing L1, L2, L3 and MD with increasing TL. While the expected trend with increasing age for radial and mean diffusivity is quite clear, there are brain regions, such as the midbrain white matter, which show decreases rather than increases in axial diffusivity [16]. Indeed, increased axial diffusivity can be interpreted as a positive marker for white matter integrity since lowered axial diffusivity indicates axon injury; by contrast, increased radial diffusivity has been linked to incomplete or damaged myelination [16]. Furthermore, association between TL and diffusion indices that are reversed compared to the effects of aging have been found in adolescent rats of the same age [18]: axial, radial and mean diffusivity were positively correlated to measured TL. In addition, FA and L1 followed inconsistent pattern in different white matter tracts when brain age was estimated [14]. Regarding the gray-white intensity contrast, previous studies demonstrated that it decreases with ageing [19, 20]. On the other hand, gray-white intensity contrast was found to be increased in people with schizophrenia and bipolar disorder compared to controls [21] although both conditions are linked to accelerated brain ageing [22–24]. In addition, increased grey-white matter contrast was observed in Autism Spectrum Disorders [25] which is also linked to accelerated brain ageing [26].

Thus, overall, this MR analysis demonstrated that the association between TL and brain IDPs is not simply the effect of increased cellular aging but there appears to be a more complex relationship underneath.

The instrumental variables used in this MR analysis involved numerous genes, which have been reported in the literature to be associated with regulating TL as well as being involved in brain disorders. The SNP rs2695242 is located within the Poly (ADP-ribose)-polymerase1 (*PARP1*) gene. *PARP1* is known to contribute largely to regulate telomere complex assembly and activity [27]. Additionally, *PARP1* was previously reported to play an essential role in neurodegenerative diseases such as AD and Parkinson’s disease [28]. In particular, it was observed that *PARP1* is activated in aging and neurodegenerative diseases leading to autophagy, neuroinflammation and mitochondrial dysfunction and dysregulation [28]. Further instrumental variable (rs7705526) belong to the Telomerase Reverse Transcriptase (*TERT*) gene. The main function of the *TERT* gene is to maintain telomeres by extending them with the telomere repeat sequence [29]. *TERT* was found to have a protective role in brain aging [30]. The authors demonstrated that neurodegenerative symptoms and brain aging were influenced by shorter telomeres, and conversely, that increasing the level of TERT in the brain of mice, and by extension the telomeres, could significantly revert signs of cognitive impairment. Lastly, the SNP rs228595 belongs to the ataxia telangiectasia mutated (*ATM*) gene which contributes telomere maintenance through telomere elongation and telomerase complex assembly [31]. It was reported that in humans, *ATM* function decreased in neurons with increasing progression of AD [32]. Thus, overall, numerous genes that harbor the instrumental variables in our MR analysis exhibit a direct role in maintaining telomeres, and have been, in previous studies, linked to degenerative brain disorders. Our study extends this link to the intermediate level of brain IDPs.

To the best of our knowledge, this is the first study to perform MR between TL and a wide range brain IDPs extracted from different MRI modalities. The results support the causal link between TL and the brain’s micro and macro structure as represented by the IDPs, with a strong emphasis on white-matter related measurements. The second finding of this study was that the direction of TL-IDP associations did not replicate the effect direction of aging-associated changes. However, the diffusion indices are influenced by multiple aspects of the brain’s micro and macro structure. For instance, despite showing a decrease in white matter integrity, increased mean diffusivity was found to be associated with increased cell density and axonal density [33]. Furthermore, current studies investigating brain age focus on linking changes in brain IDPs with genetic variations and environment factors [34], but have not considered the potentially driving role of TL. To summarize, our study showed that TL significantly influenced 193 IDPs covering diffusion MRI metrics, cortical grey-white contrast regions, resting state fMRI and morphometric measures making TL a valuable feature to be considered when estimating and investigating brain age.

## Materials and methods

### TL GWAS

We selected 33 SNPs from different publicly available TL GWAS studies [1, 6]. The first 20 SNPs at 17 genomic loci were from the recent GWAS by Li et al. [6]. They conducted a large-scale GWAS in up to 78,592 European individuals, under the ENGAGE project (European Network for Genetic and Genomic Epidemiology). Polymerase Chain Reaction (PCR) technique was established to measure mean leukocyte TL quantitatively. The TL was presented as the ratio of the telomere repeat number to a single-copy gene. Sex, age and cohort-specific factors including genetic principle components and center were used as covariates in the GWAS. The selected 20 SNPs were significantly and independently associated with leukocyte TL. However, six SNPs were substituted to their proxies as they were palindromic [8] (S2 Table for details). For that purpose, LDlink was used to select suitable proxies [35].

The remaining 13 SNPs were used previously by Kuo et al. [1] to perform an MR analysis between TL and aging-related diseases in 261,000 older participants in the United Kingdom Biobank (UKB). The authors selected SNPs that were significantly (*P* < 5×10^−8^) associated with TL from previous GWAS studies. The SNPs used in their study included GWAS results from [7] using European population and six GWAS comprising 9,190 European participants [36]. We added these 13 SNPs to the previously selected 20 SNPs. Ten SNPs were removed because they were in high linkage disequilibrium (LD) with other SNPs (*R*^2^ > 0.02). LD was calculated using GBR (British in England and Scotland) samples from Phase 3 (version 5) of the 1,000 Genomes Project using Ensembl 2020 [37]. The final list for our study comprised 23 SNPs (listed in Table 2).

**Table 2:** List of the SNPs used in the MR analysis. rsID, ID of the SNP; Chr, chromosome; Pos, position of the SNP in the genome; EA, effect allele; OA, other allele; EAF, effect allele frequency; Beta, beta value of the SNP in GWAS; SE, standard error.

rsID	Chr	Pos	Gene	EA	OA	EAF	Beta	SE	P-value	Source
rs2695242	1	226594038	PARP1	G	T	0.83	-0.039	0.0064	9.31E-11	[6]
rs11125529	2	54475866	ACYP2	A	C	0.16	0.065	0.012	4.48E-08	[7]
rs6772228	3	58376019	PXK	T	A	0.76	0.041	0.014	3.91E-10	[38]
rs55749605	3	101232093	SENP7	A	C	0.58	-0.037	0.007	2.45E-08	[6]
rs7643115	3	169512241	TERC	A	G	0.243	-0.0858	0.0057	6.42E-51	[6]
rs13137667	4	71774347	MOB1B	C	T	0.959	0.0765	0.0137	2.37E-08	[6]
rs7675998	4	164007820	NAF1	G	A	0.8	0.048	0.012	4.35E-16	[7]
rs7705526	5	1285974	TERT	A	C	0.328	0.082	0.0058	4.82E-45	[6]
rs34991172	6	25480328	CARMIL1	G	T	0.068	-0.0608	0.0105	6.03E-09	[6]
rs805297	6	31622606	PRRC2A	A	C	0.313	0.0345	0.0055	3.41E-10	[6]
rs59294613	7	124554267	POT1	A	C	0.293	-0.0407	0.0055	1.12E-13	[6]
rs9419958	10	105675946	STN1 (OBFC1)	C	T	0.862	-0.0636	0.0071	4.77E-19	[6]
rs228595	11	108105593	ATM	A	G	0.417	-0.0285	0.005	1.39E-08	[6]
rs76891117	14	73399837	DCAF4	G	A	0.1	0.0476	0.0084	1.64E-08	[6]
rs3785074	16	69406986	TERF2	G	A	0.263	0.0351	0.0056	4.5E-10	[6]
rs62053580	16	74680074	RFWD3	G	A	0.169	-0.0389	0.0071	3.96E-08	[6]
rs7194734	16	82199980	MPHOSPH6	T	C	0.782	-0.0369	0.006	6.72E-10	[6]
rs3027234	17	8136092	CTC1	C	T	0.83	0.103	0.012	2E-08	[36]
rs8105767	19	22215441	ZNF208	G	A	0.289	0.0392	0.0054	5.21E-13	[6]
rs6028466	20	38129002	DHX35	A	G	0.17	0.058	0.013	2.57E-08	[6, 36]
rs71325459	20	62268341	RTEL1	T	C	0.015	-0.1397	0.0227	7.04E-10	[6]
rs75691080	20	62269750	STMN3	T	C	0.091	-0.0671	0.0089	5.75E-14	[6]
rs73624724	20	62436398	ZBTB46	C	T	0.129	0.0507	0.0074	6.08E-12	[6]

### GWAS for brain IDPs

To represent brain IDPs, we used publicly available GWAS summary statistics of 3,935 brain IDPs [39] (https://open.win.ox.ac.uk/ukbiobank/big40/). Briefly, the results are based on 33,000 subjects (22,000 in discovery and 11,000 in replication) from UKB and the IDPs covered six MRI modalities (T1-weighted MRI, T2-FLAIR, susceptibility-weighted MRI, diffusion MRI, task and resting-state functional MRI). They conducted GWAS for recent UK ancestry based on genetic principal component and self-reported ancestry. In addition, they excluded related participants to consider only unrelated individuals. Furthermore, they included the X-chromosome in their analysis. 597 variables were used as confounds including age, sex, head motion, head size and genetic principal components. More details about the quality control and the used data can be found in [39].

### Ethics statement

This study is based on Mendelian randomization using publicly available summary statistics of genome wide association studies. Since these summary statistics are fully anonymised, no ethics approval was required.

## Statistical analysis

The TL GWAS included participants from different cohorts such EPIC-CVD and the EPIC-InterAct case-cohort study which was conducted in ten countries including UK [6]. On the other hand, GWAS for brain IDPs was conducted on majority healthy [13] participants (at recruitment time) only from UK. There is a low possibility of overlapping participants between the TL GWAS and the brain IDP GWAS. We conducted MR analysis using the TwoSampleMR [40] package in R. For each brain IDP, we first downloaded the GWAS results and extracted the beta value, standard error, effect allele, other allele, effect allele frequency and *p*-value for each SNP selected from TL GWAS studies (Table 2). Then we harmonised the data from TL GWAS and the brain IDPs GWAS using the harmonise_data() function. Finally, we performed MR between TL and brain IDPs. The (random effects) IVW method was adopted as a primary analysis for SNP-specific casual estimate for brain IDPs. *P*-values were corrected for multiple tests using the false discovery rate (FDR) method [41]. IDPs were considered significant at *P*_FDR_ < 0.05 (corresponding to *P* < 0.004409). Weighted median and weighted mode approaches were also implemented as complementary MR analyses (requiring uncorrected *P* < 0.05). To detect directional pleiotropy and heterogeneity of the genetic instruments, weighted median function [42] and MR-Egger [43] regression were performed. The MR-Egger intercept test (*P* > 0.05), leave-one-SNP-out analyses and the modified Cochran Q statistic methods were implemented as horizontal pleiotropy test and to assess the quality of results. In addition, we used MR–Pleiotropy Residual Sum and Outlier (MR-PRESSO) [9] to detect and correct pleiotropy which affected the overall results. Thus, for IDPs surviving our filtering by IVW (*P*_FDR_ < 0.05) and complementary analyses (*P* < 0.05), we retained IDPs when they showed either no horizontal pleiotropy in the MR-PRESSO global test (*P* > 0.05) or the IVW adjusted for SNP outliers detected by MR-PRESSO remained significant (*P*_FDR_ < 0.05). For each brain IDP association with TL, we generated an html file report using the command mr_report() in the package. The html file contains on all the results of the methods mentioned earlier.

## Supporting information

S1 Table Full list of all MR results. (XLSX)

S2 Table Information on SNPs and their selected proxies for the MR analysis. (DOCX)
