# Supplementary material for: Telomere length is causally connected to brain MRI image derived phenotypes: A mendelian randomization study
Source: PLoS One. 2022 Nov 18;17(11):e0277344. doi: 10.1371/journal.pone.0277344 (PMC9674175; doi:10.1371/journal.pone.0277344)
Supplement: S2 Table — (DOCX) [file pone.0277344.s002.docx]

**Table S2: Information on SNPs and their selected proxies for the MR analysis.**

| **Original** | | | | | | | **Proxy** | | | | | |
| --- | --- | --- | --- | --- | --- | --- | --- | --- | --- | --- | --- | --- |
| **Locus** | **SNP** | **Chr** | **Position** | **EA** | **OA** | **MAF (CEU+GBR)** | **SNP** | **Position** | **EA** | **OA** | **LD (r2)** | **MAF (CEU+GBR)** |
| *PARP1* | rs3219104 | 1 | 226562621 | C | A | 0.147 | rs2695242 | 226594038 | G | T | 0.98 | 0.15 |
| *TERC* | rs10936600 | 3 | 169514585 | T | A | 0.261 | rs7643115 | 169512241 | A | G | 1 | 0.261 |
| *NAF1* | rs4691895 | 4 | 164048199 | C | G | 0.232 | rs7675998 | 164007820 | G | A | 0.93 | 0.229 |
| *PRRC2A* | rs2736176 | 6 | 31587561 | C | G | 0.284 | rs805297 | 31622606 | A | C | 1 | 0.284 |
| *DCAF4* | rs2302588 | 14 | 73404752 | C | G | 0.111 | rs76891117 | 73399837 | G | A | 1 | 0.111 |
| *RTEL1* | [rs34978822](https://www.sciencedirect.com/science/article/pii/S0002929720300483#tblfn1) | 20 | 62291599 | G | C | 0.021 | rs71325459 | 62268341 | T | C | 1 | 0.021 |

Chr = chromosome; Positions = genome position based on human genome release hg19; EA= Effect Allele; OA = Other allele; MAF = minor allele frequency in Central Europeans (CEU) and British ancestry (GBR) in the 1000 genomes dataset; LD = linkage disequilibrium between the original SNP and the proxy SNP expressed in *r^2^* in Central Europeans and British ancestry.
